# Supplementary material for: Structural mechanism of intracellular autoregulation of zinc uptake in ZIP transporters
Source: Nat Commun. 2023 Jun 9;14:3404. doi: 10.1038/s41467-023-39010-6 (PMC10256678; doi:10.1038/s41467-023-39010-6)
Supplement: Supplementary file 1 — Supplementary Information [file 41467_2023_39010_MOESM1_ESM.pdf]

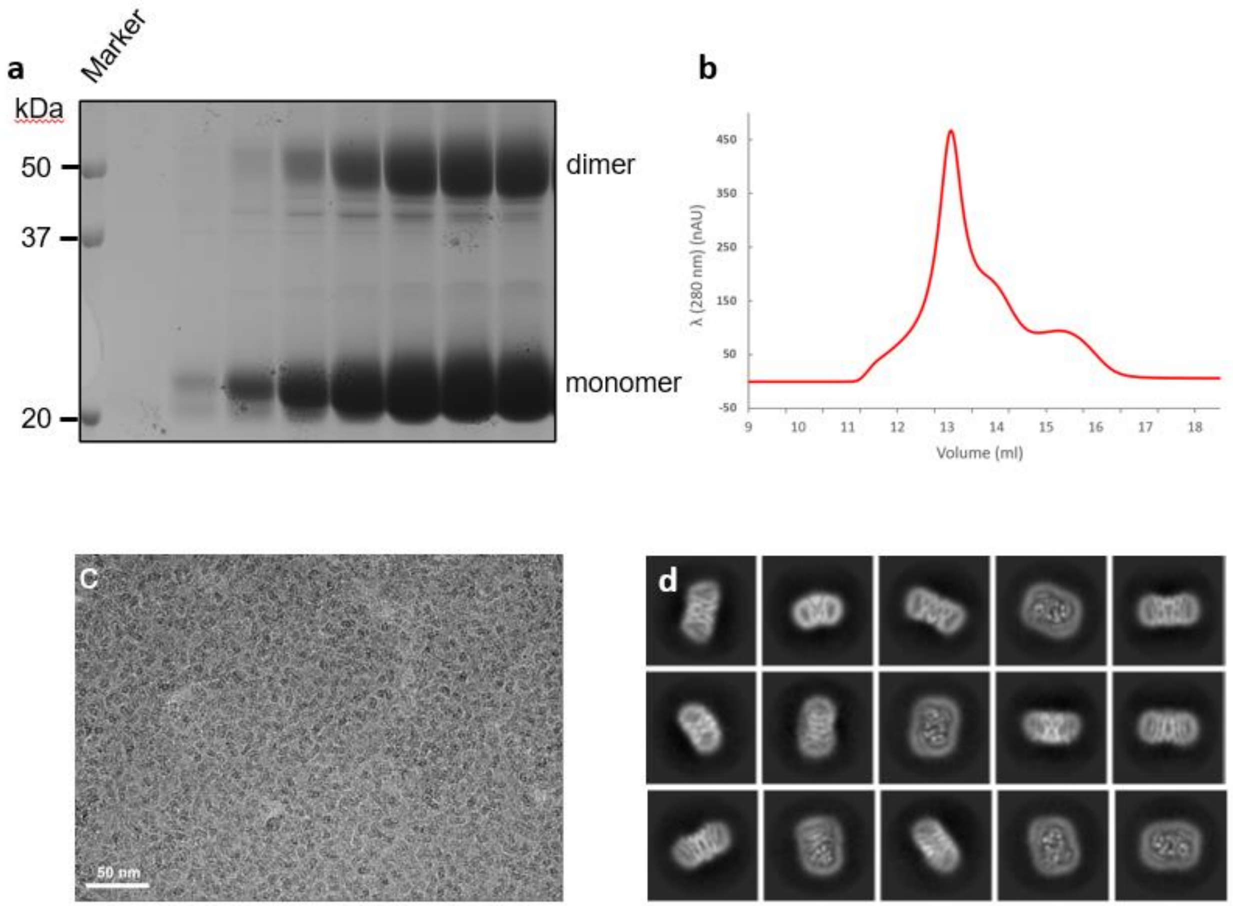

Supplementary Fig. 1 Protein production and cryo-EM sample preparation. **a**, SDS-PAGE analysis for the purified *Bb*ZIP. The figure represents at least three independent experiments with similar results. The lanes are eluted fractions from initial size-exclusion chromatography. **b**, Size-exclusion chromatography (SEC) peak profile of the purified protein reconstituted in amphipol PMAL-C8. **c**, A typical motion-corrected cryo-EM micrograph from a total of 6909 micrographs. Each particle is a 2D projection and alone does not provide adequate resolution for a high-resolution 3D structure. **d**, Selected views of 2D averaged particles.

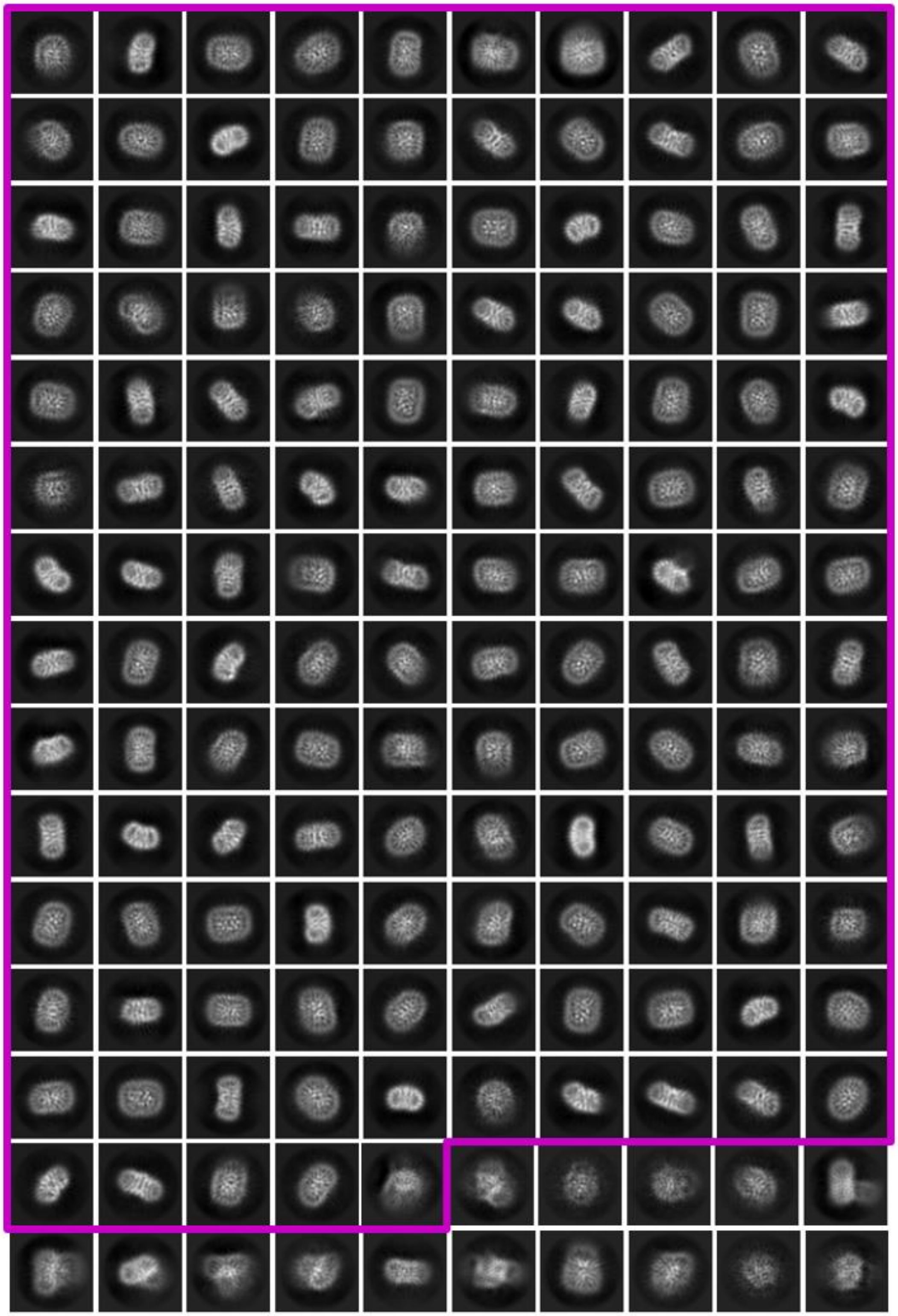

Supplementary Fig. 2 Classification of particles by 2D averaging. Particles were 2D class averaged using cryoSPARC with 150 classes. The 135 classes selected for the generation of *ab initio* models are indicated by using a magenta contour.

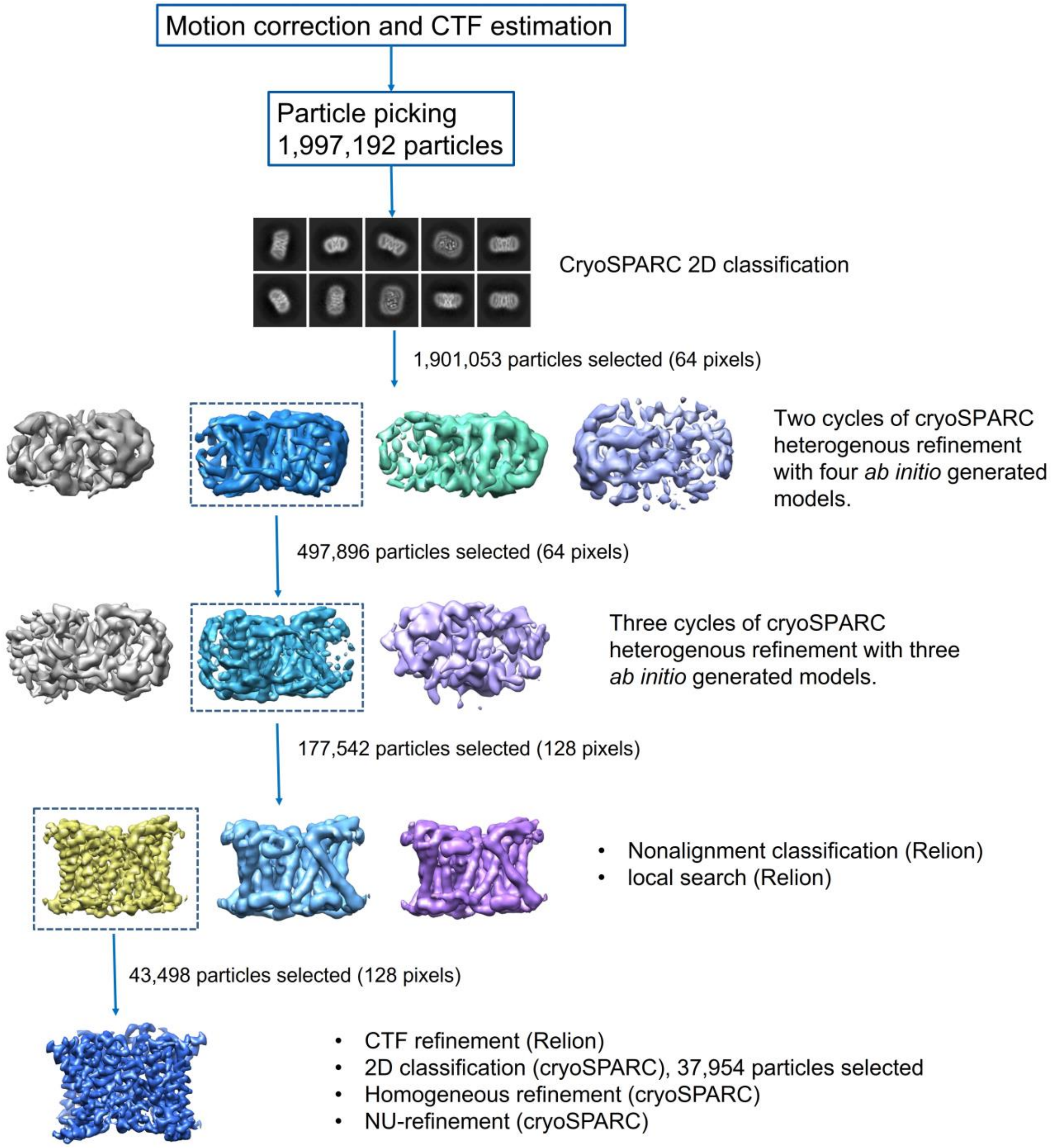

Supplementary Fig. 3 A data analysis workflow for the reconstruction of the *Bb*ZIP dimer.

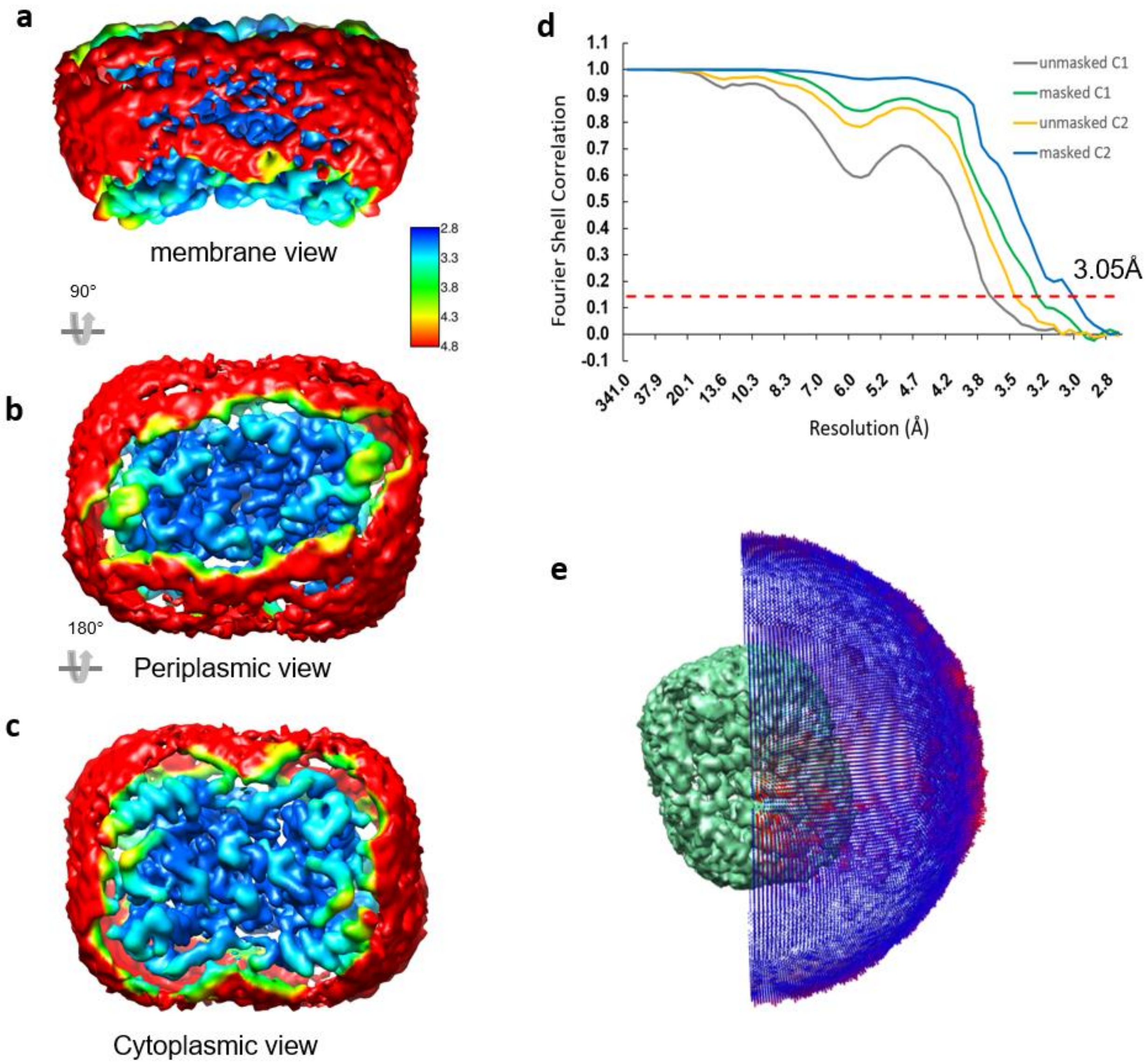

Supplementary Fig. 4 Structure determination analysis. **a-c**, Three views for the reconstructed map colored with local resolutions. **d**, Fourier Shell Correlation (FSC) curves for the unmasked and masked 3D reconstruction to determine the resolution with two-fold symmetry (C2) or no symmetry (C1). **e**, Orientation distribution for particles used in the 3D reconstruction.

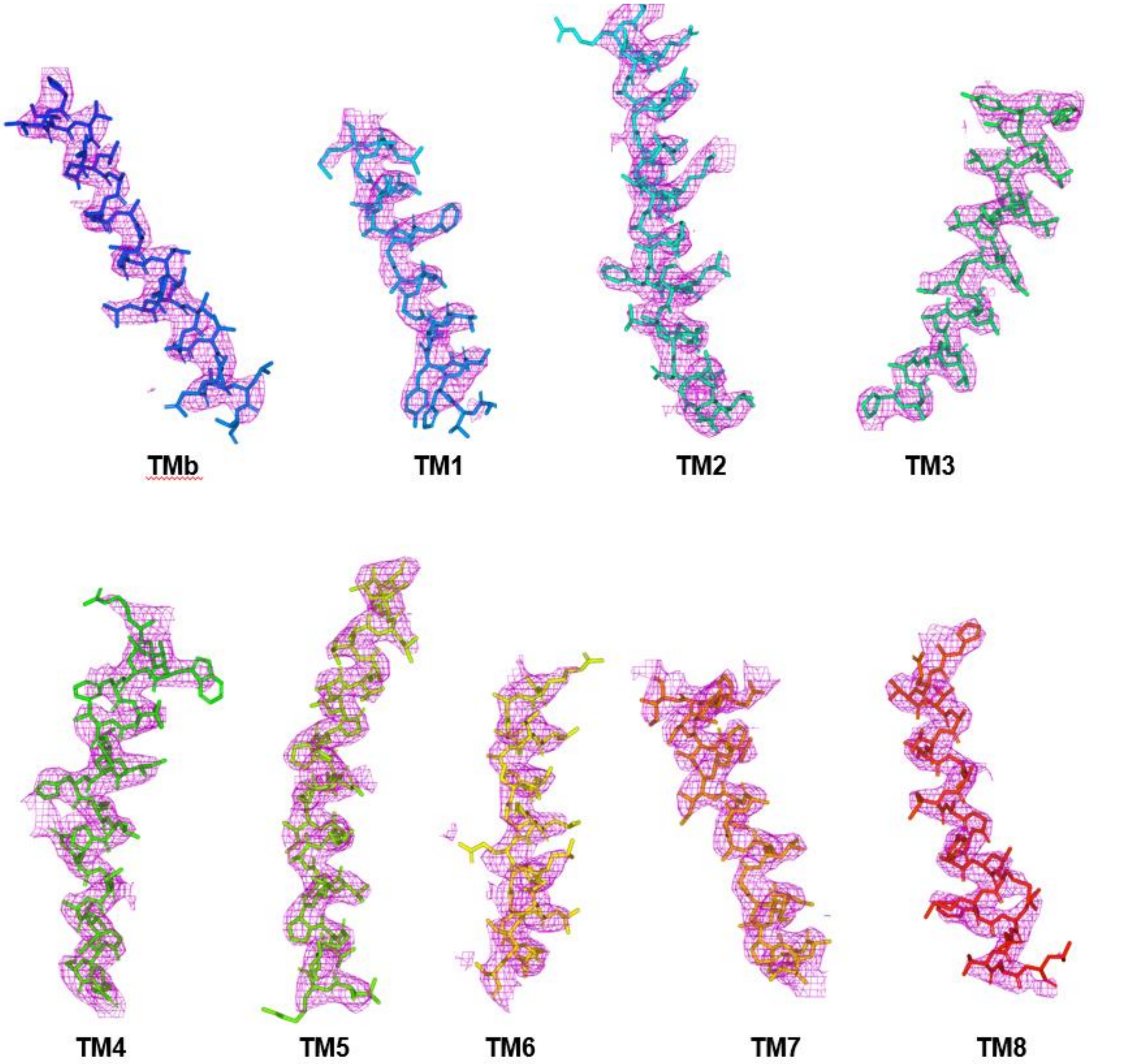

Supplementary Fig. 5 Cryo-EM densities for the nine transmembrane helices in a *Bb*ZIP monomer to show the quality of the map. The densities are shown as magenta isomeshes contoured at 7σ. Transmembrane helices are shown as sticks.

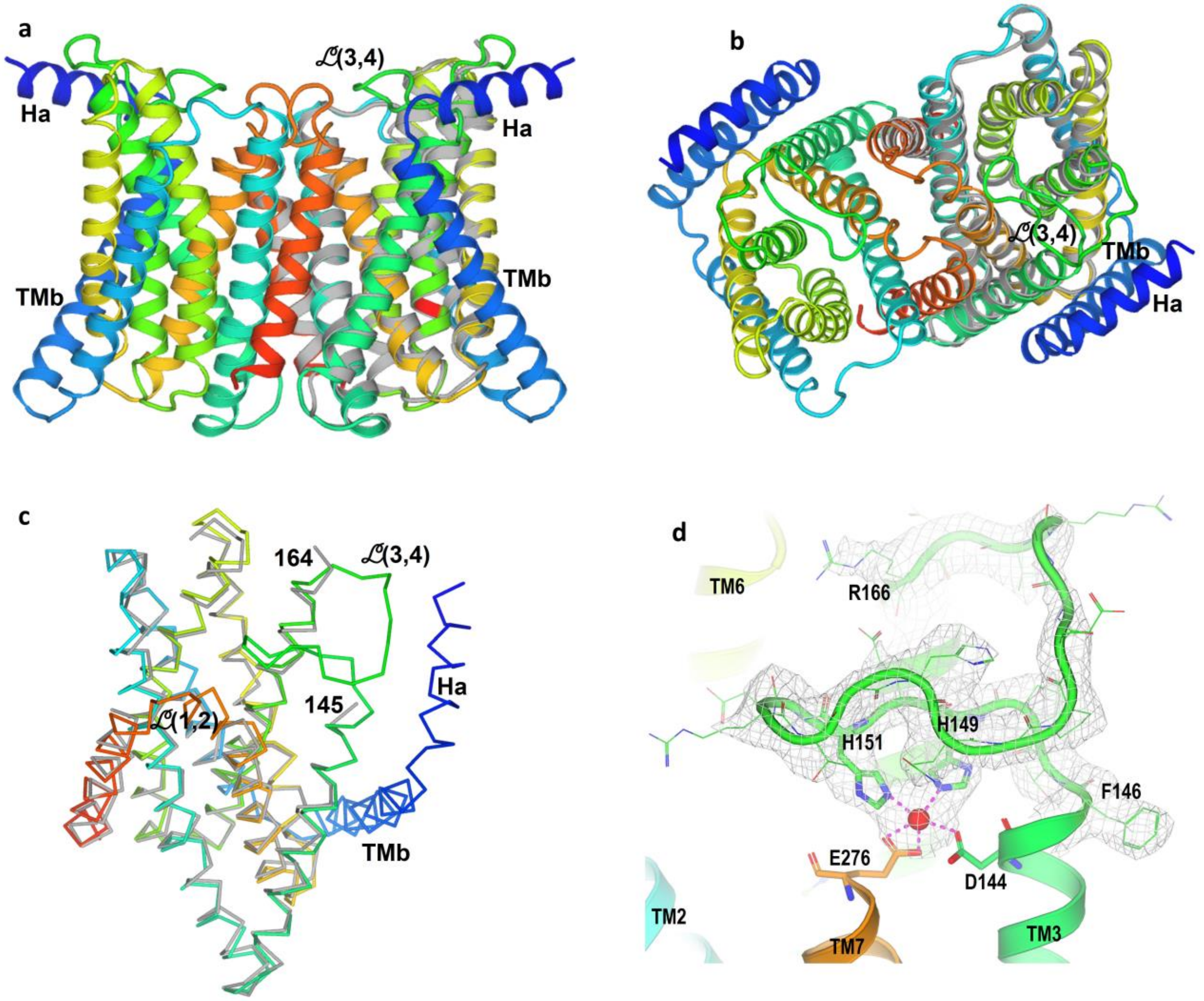

Supplementary Fig. 6 Structural comparisons of *Bb*ZIP dimer with its crystal structure. a-b, Two cartoon views of aligned *Bb*ZIP dimer (rainbow color with N-terminal blue and C-terminal red) and the crystal structure (gray). **c**, A ribbon view of the aligned *Bb*ZIP protomer to highlight the three regions of difference in the cryo-EM (rainbow color) and crystal structure (gray). The three regions were labeled as Ha and TMb, ℒ(1,2), and loop ℒ(3,4). **d**, Cryo-EM density for the loop ℒ(3,4), covering residues from F146 to R166. The coloring is the same as **c**. Metal ion Cd^{2+} is shown as a red sphere.

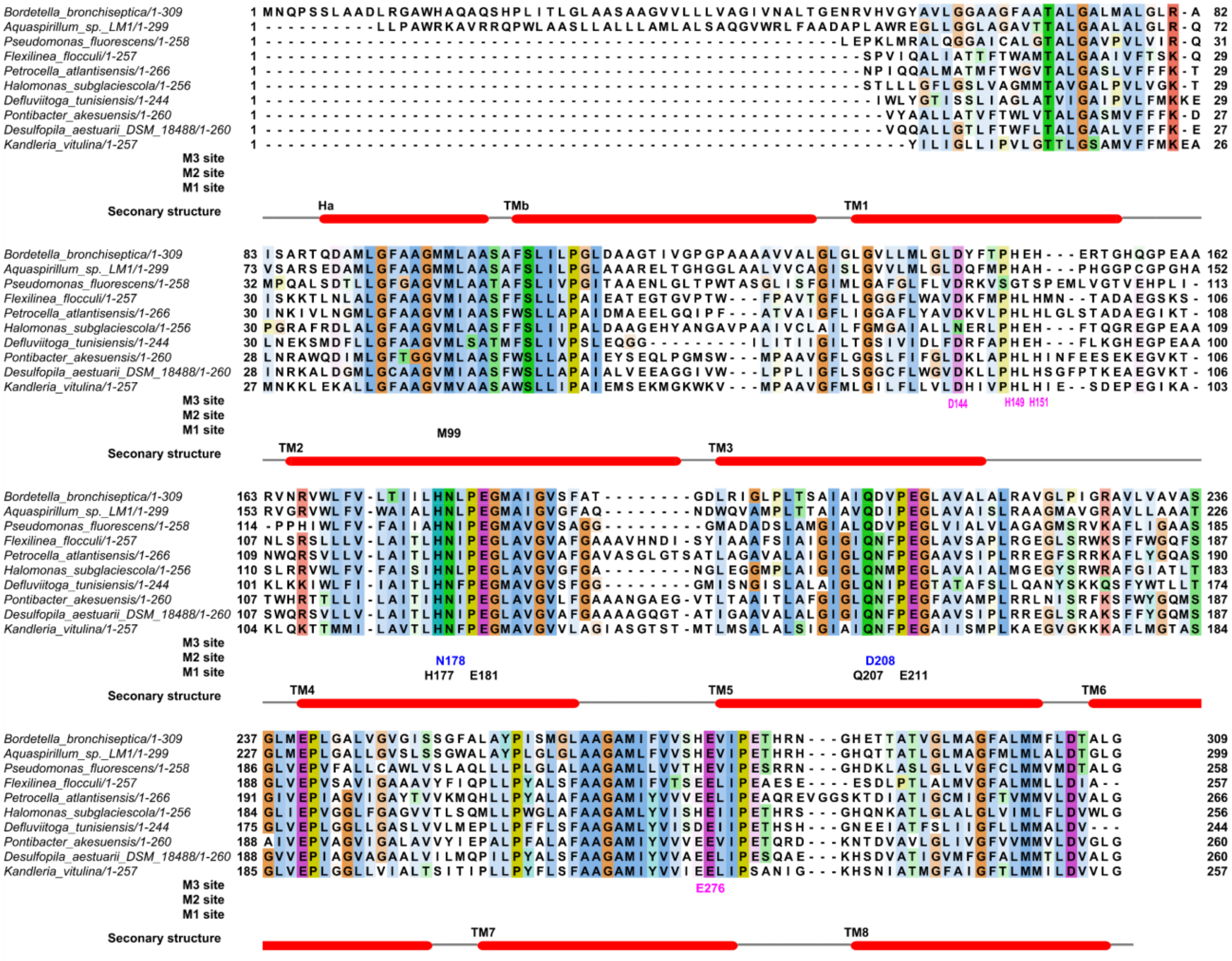

Supplementary Fig. 7 Sequence alignments of ZIPs. Residues defining the M1, M2, and M3 sites are indicated and colored differently. The residue numbering is for *Bb*ZIP (*Bordetella bronchiseptica*)**.** Uniprot numbers used for sequence alignments are A0A0H3LM39 from *Bordetella bronchiseptica*, A0A1U9JI25 from *Aquaspirillum_sp._LM1*, A0A5E6W282 from *Pseudomonas fluorescens*, A0A0S7BU43 from *Flexilinea flocculi*, A0A3P7PD27 from *Petrocella atlantisensis*, A0A1M7I1H2 from *Halomonas subglaciescola*, A0A0C7NZ34 from *Defluviitoga tunisiensis*, A0A1I7GPW4 from *Pontibacter akesuensis*, A0A1M7YB74 from *Desulfopila aestuarii DSM 18488*, and A0A1H2R0P4 from *Kandleria vitulina.*

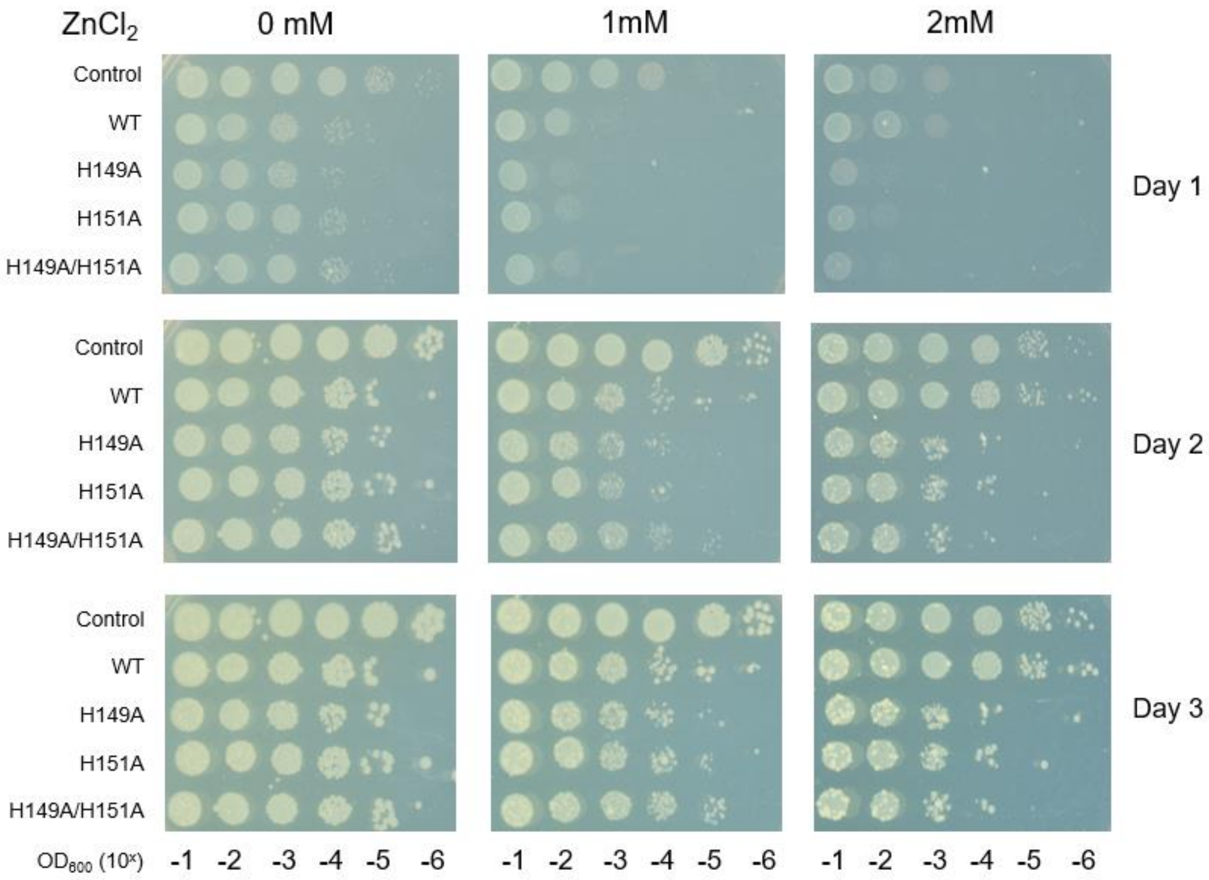

Supplementary Fig. 8 Cell growth viability assays of *Bb*ZIP wild-type and histidine mutants in the loop ℒ(3,4). *E. coli* C43 cells transformed with indicated plasmids for plating Agar-LB plates with indicated $ZnCl_2$ and presence of 0.2 mM IPTG (final). Before plating, cells were diluted to indicated OD_{600} values. "x" indicates the dilution factor from 10^{-1} to 10^{-6}. The cell growth was monitored on day 1, day 2, and day 3. Compared with the control which has an empty plasmid, histidine mutants in the loop showed increased cell toxicity with externally added Zn^{2+} concentration at 1 or 2 mM.

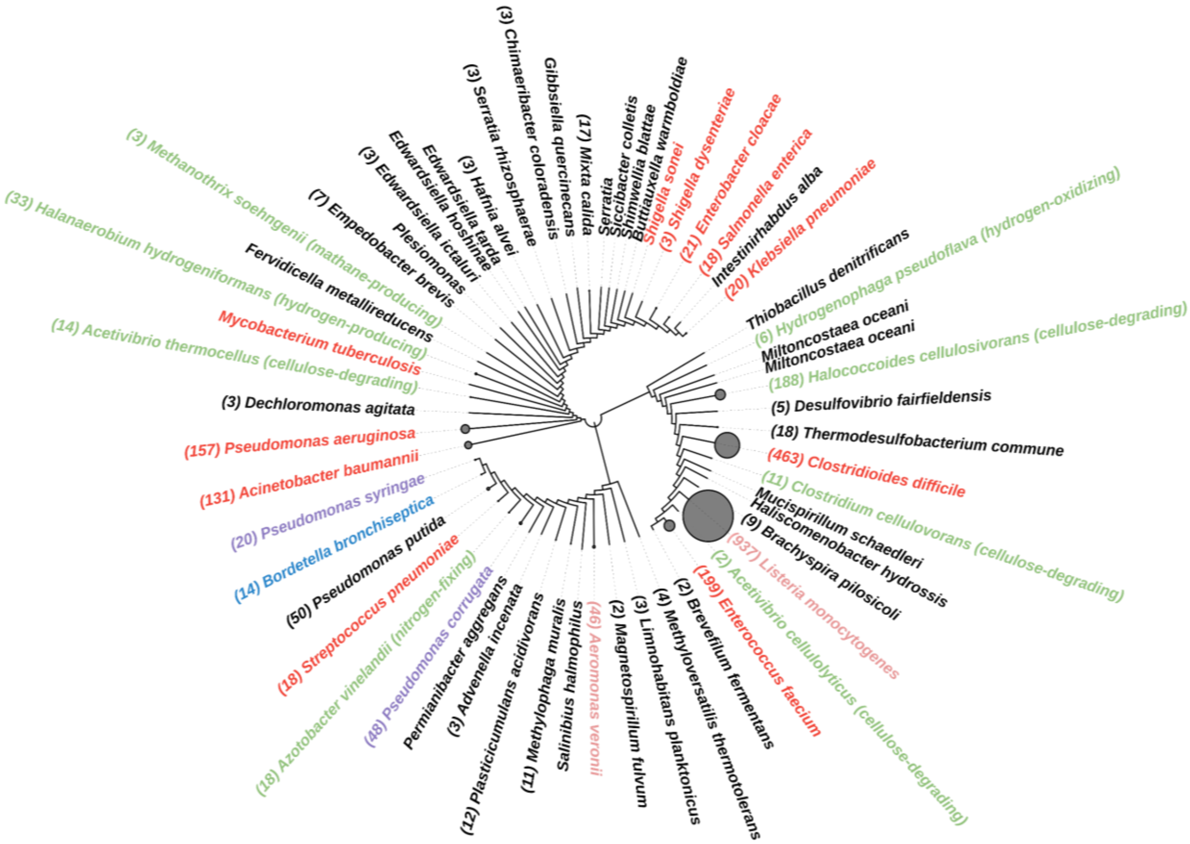

Supplementary Fig. 9 A cladogram of ZIPs from bacteria and archaea. The multiple sequence alignment and neighbor-joining tree were conducted in MEGA11 with default settings [60]. The number in the bracket indicates the number of leaves of that branch. Human pathogens are colored in red; plant pathogens are colored in violet; and microorganisms of industrial interests are colored in green; *Bordetella bronchiseptica* is colored in blue.

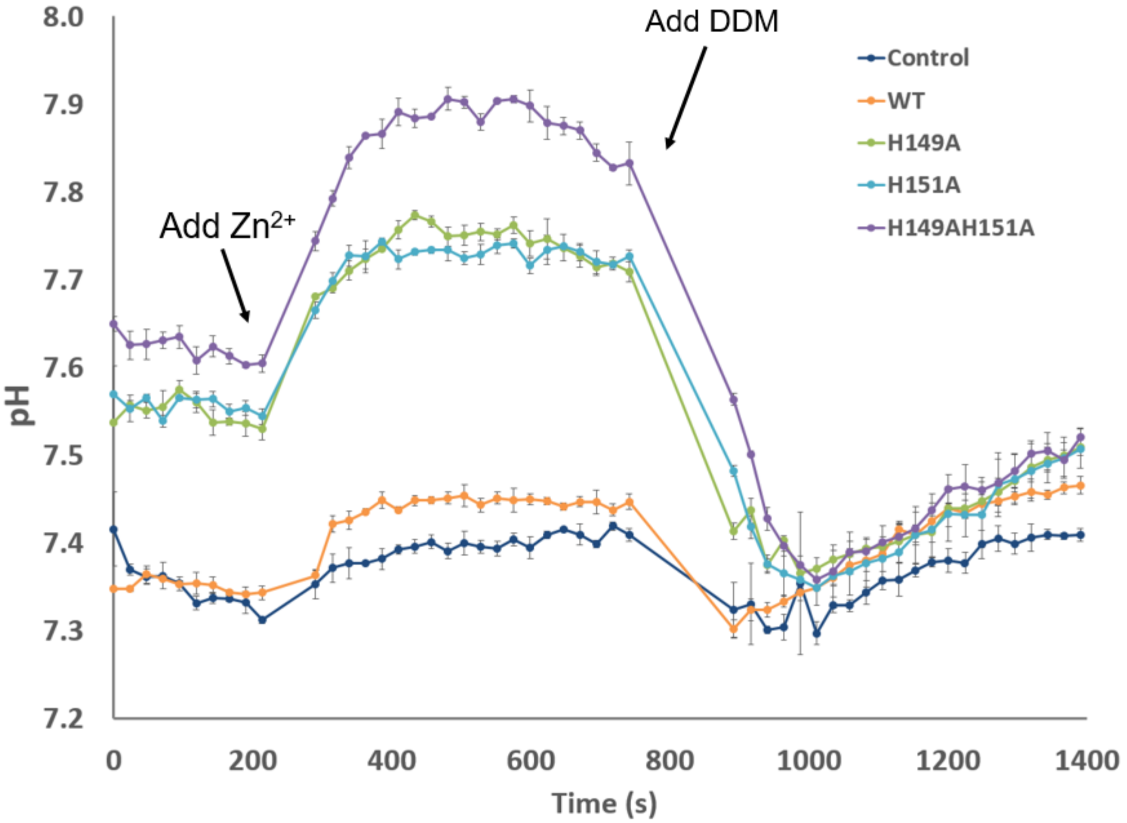

Supplementary Fig. 10 Zn^{2+} uptake by *Bb*ZIP induced intracellular pH change. Cells transformed by indicated plasmids were used for Zn^{2+} uptake assay using preloaded BCECF. With externally added Zn^{2+} to a final concentration of 500 μM at pH 7.5, intracellular fluorescence intensities normalized to OD_{600} were measured. Data are presented as mean values ± SD. The error bar is the standard deviation (s.d.) derived from three different samples. The experiments were replicated at least three times.

Supplementary Table 1 Cryo-EM data collection, 3D reconstruction, and refinement statistics.

**Data Collection**	
Microscope	Titan Krios G3i
Magnification	130 Kx
Stage type	Autoloader
Voltage (kV)	300
Detector	Gatan K3
Energy filter (eV)	15
Acquisition mode	Super-resolution
Physical pixel size (Å)	0.666
Defocus range (µm)	0.7-2.5
Electron exposure (e^-/Å^2)	64
**Reconstruction**	
Software	Relion v3.08, cryoSPARC v2.15
Particles picked	1,997,192
Particles final	37,954
Extraction box size (pixels)	256
Rescaled box size (pixels)	64
Final pixel size	1.332
Symmetry imposed	C2
Map resolution (Å)	3.05
FSC threshold	0.143
Map resolution range (Å)	341-2.77
Map sharpending B-factor (Å^2)	41
**model refinement**	

Software	PHENIX
Refinement algorithm	Real Space
Clipped box size (pixels)	None
Number of residues	608
Number of Cadmium	6
Number of lipids	2
R.m.s deviations	
Bond length (Å)	0.004
Bond angle (°)	0.641
Molprobity clashscore	5.60
Rotamer outliers (%)	0.0
Cβ deviations (%)	0.0
Ramachandran plot	
Favored (%)	93.05
Allowed (%)	6.62
Outliers (%)	0.33
PDB code	8GHT
