# Supplementary material for: Structural mechanism of intracellular autoregulation of zinc uptake in ZIP transporters
Source: Nat Commun. 2023 Jun 9;14:3404. doi: 10.1038/s41467-023-39010-6 (PMC10256678; doi:10.1038/s41467-023-39010-6)
Supplement: Supplementary file 4 — Source Data [file 41467_2023_39010_MOESM4_ESM.zip › 10.1038-s41467-023-39010-6 - NCOMMS-22-44922/liuetalfiles.zip!/zipfile/uncropped gel to be zipped.pdf]

Uncropped supplementary Fig. 1a

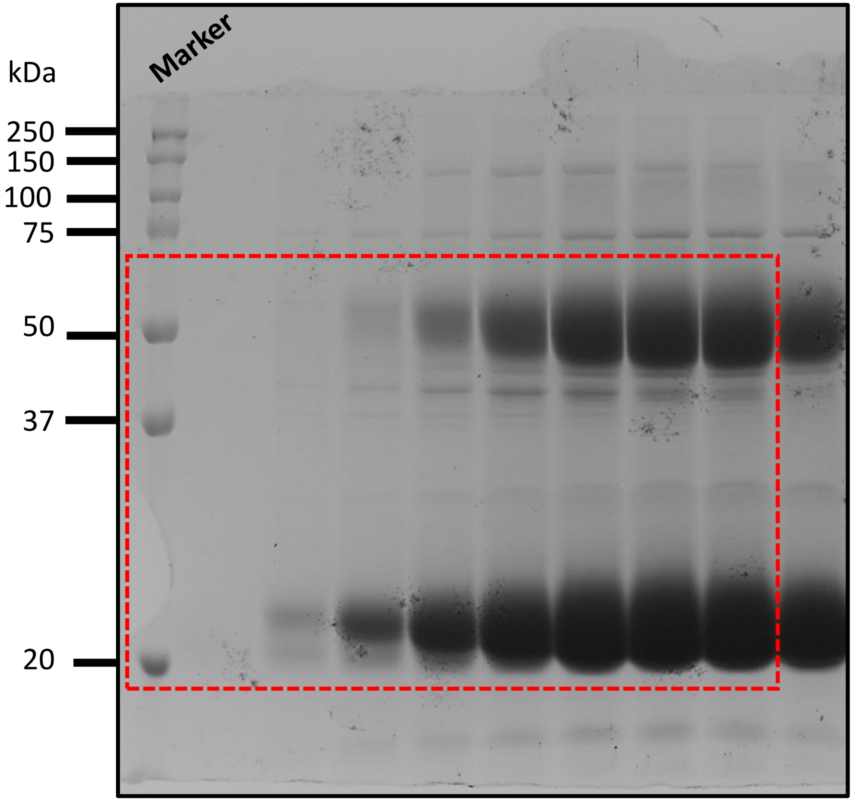

Supplementary Fig. 1a in the manuscript is marked in a dashed red rectangle.
